# Disulfidptosis-related prognostic signature correlates with immunotherapy response in colorectal cancer

**DOI:** 10.1038/s41598-023-49954-w

**Published:** 2024-01-02

**Authors:** Yu Xiao, Hancui Lin, Jinluan Li, Junxin Wu

**Affiliations:** Department of Radiation Oncology, Clinical Oncology School of Fujian Medical University, Fujian Cancer Hospital, Fuzhou, 350014 China

**Keywords:** Cancer, Computational biology and bioinformatics, Oncology

## Abstract

Disulfidptosis (DSP), a form of cell death caused by disulphide stress, plays an important role in tumour progression. However, the mechanisms by which DSP regulates the tumour microenvironment remain unclear. Thus, we analysed the transcriptome profiles and clinical data, which were obtained from the TCGA database, of 540 patients with colorectal cancer. Compared with the patients with low DSP expression, those with high DSP expression exhibited significantly better survival outcomes; lower stromal and ESTIMATE scores; significantly higher numbers of CD4+ T cells, M2 macrophages, dendritic cells, and neutrophils; higher expression of immune checkpoint-related genes; and lower Tregs and HLA-DQB2 levels. A prognostic signature established based on DSP-related genes demonstrated an increase in risk score with a higher clinical stage. Risk scores negatively correlated with dendritic cells, eosinophils, and CD4+ T cells and significantly positively correlated with Treg cells. Patients with higher risk scores experienced significantly worse survival outcomes and immunotherapy non-response. Our nomogram model, combining clinicopathological features and risk scores, exhibited robust prognostic and predictive power. In conclusion, DSP-related genes actively participated in regulating the tumour microenvironment. Thus, they can serve as biomarkers to provide targeted treatment for colorectal cancer.

## Introduction

Colorectal cancer (CRC) represents a significant global health challenge^1^, with 153,020 new cases and 52,550 deaths reported in the United States in 2023, ranking it third in terms of incidence and mortality^2,3^. CRC is difficult to treat and has poor prognosis because it is highly invasive and metastatic and prone to treatment resistance. With the development of precision therapy, research into targets such as BRAF, HER2, RAS, NTRK, and VEGF has progressed. Consequently, CRC treatment has gradually entered the stage of diversified precision therapy^4–7^. However, CRC responding to targeted therapy eventually become prone to drug resistance^8,9^. Thus, novel targets need to be explored to improve the prognosis of patients with CRC. The development of an effective risk-based prognostic model will make targeted therapy more feasible.

Cell death, similar to cell proliferation and differentiation, contributes to the maintenance of biological life^10,11^. Cancer cells, in their pursuit of unlimited proliferation, often employ various mechanisms to evade death. Therefore, inducing programmed cell death has emerged as an important strategy in tumour treatment. Common mechanisms of cell death include apoptosis, necroptosis, pyroptosis, ferroptosis, and autophagy-dependent cell death. Recently, researchers have discovered a new cell death mechanism, disulfidptosis (DSP), which provides fresh insights into developing cancer treatment strategies^12^. DSP involves the SLC7A11 protein, a solute carrier family member which facilitates the transport of nutrients and metabolites across the cell membrane^13^. SLC7A11 transports extracellular cystine to intracellular cells and glutamate from the intracellular to the extracellular^14^. Unlike healthy cells, cancer cells are highly dependent on cystine input. SLC7A11 is overexpressed in many cancers, including lung and kidney cancers^15–17^. Each cysteine molecule which enters the cell is reduced to two cysteine residues by the consumption of NADPH molecules. Under glucose starvation, SLC7A11-overexpressing cells have reduced NADPH levels, which prevent the reduction of cysteine to cystine. As a result, cysteine and other disulfide compounds accumulate in the cells, leading to disulfide stress and rapid cell death.

DSP plays a crucial role in the development of malignant tumours. During the occurrence and development of malignant tumours, various forms of cell death are often overcome^18^. However, the relationship between DSP and CRC remains unclear. In addition, the identification of genes predicting survival prognosis is essential to guiding personalised treatment for patients with CRC. A recent study by Liu and colleagues used CRISPR-Cas9 libraries to screen multiple biomarkers and hinted at therapeutic strategies for targeting disulfide stress in cancer therapy^12^. Therefore, this study aimed to establish a novel DSP-related gene risk prognostic signature to predict the treatment outcomes and prognosis of patients with CRC. It also assessed heterogeneity among patients with CRC and evaluated their clinical prognosis. The findings of this study may guide the selection of appropriate treatment options.

## Materials and methods

### Data collection

The research process is illustrated in Fig. S1. Firstly, we obtained transcriptome data from The Cancer Genome Atlas database (TCGA, https://portal.gdc.cancer.gov/) for 568 patients with CRC, of which 28 patients had incomplete clinical data and were excluded. Therefore, those patients with complete information (n = 540) were included in our training dataset for further analysis. In addition, we further tested the accuracy of the prognostic signature by downloading GSE39582 data from the Gene Expression Omnibus (GEO) (https://www.ncbi.nlm.nih.gov/geo/) database as a validation set, which included 585 patients with CRC with complete transcriptional, clinicopathological information. Baseline information on clinicopathological features were shown in Table S1. Perl software is used for data sorting and ID conversion of transcriptome data.

### Identification of differentially expressed genes in cancer and normal tissues

DSP-related genes were sourced from a previous study^12^. Multiple biomarkers were screened using the CRISPR-Cas9 library, and 902 DSP-related genes with *P* < 0.05 were included in this study. Subsequently, we used the free software programming R language for statistical analysis, graphics, and data mining. R packages are collections of functions and data sets developed by the community^19^. The R package ‘limma’ was used to distinguish differentially expressed genes (DEGs) in TCGA patients with CRC (*P* < 0.05)^20^.

### Consensus clustering analysis of DSPs

The identification of cancer molecular subtypes is important for targeted therapy and tumour classification and stratification. We used the R package ‘ConsensusClusterPlus’ for unsupervised cluster analysis to explore CRC DSP subtypes^21^. To explore the potential differences between different DSP molecular subtypes, we used subtype differential genes for follow-up analysis. The R package ‘limma’ was used to find DEGs among different DSP subtypes^20^. Fold-change > 1 and *P* < 0.05 were the criteria for selecting DEGs. Gene Ontology (GO) and Kyoto Encyclopedia of Genes and Genomes (KEGG) were used for DEG function enrichment^22–25^. To further explore whether DSP is involved in regulating the immune microenvironment of CRC, R packages ‘CIBERSORT’ and ‘ESTIMATE’ were further utilized^26–28^. Based on the linear support vector regression principle, CIBERSORT is a tool for deconvoluting the expression matrix of human immune cell subtypes using transcriptome sequencing data to calculate the proportion of different cell types^27^. ESTIMATE requires the transcription profile of cancer samples to infer the number of tumour cells, as well as the number of infiltrating immune cells and stromal cells^28^. Further, the R package ‘limma’ was used to analyze the difference in immune cells and scores between different DSP subtypes^20^. It was also used to analyze the differential expression of HLA and immune checkpoint-related genes which were collected from previous studies.

### Construction and validation of DSP-related gene prognostic signature

Univariate Cox regression analysis was used to identify the prognostic DSPs in CRC. The TCGA COAD/READ cohort served as the training set. Least absolute shrinkage and selection operator (LASSO) Cox regression analysis was used to construct a prognostic signature^29^. The risk of overfitting was minimized by executing the ‘glmnet’ function in the R package^30^. Subsequently, risk scores were calculated based on the mRNA expression of prognostic signature genes.

The formula of the risk score is as follows:

$$\mathrm{Risk\, scores}= \sum_{i=1}^{n}(\mathrm{gene\, expression}\times {\text{coefficient}})$$

The GEO database GSE39582 was used as an external dataset for signature validation. Patients with CRC in the training and testing sets were divided into high- and low-risk groups, based on the median risk score of the training set. The R package ‘survminer’ was used to evaluate the overall survival (OS) between the high- and low-risk subgroups.

### Analysis of DSP risk score and clinicopathological features

The R packages ‘limma’ was used to analyse the relationship between DSP risk score and clinicopathological features, including age, sex, T, N, M, TNM stage^20^.

### Analysis of the relationship between DSP risk score and immune microenvironment

CIBERSORT algorithm was further used for analysing the correlation between DSP risk score and immune cell infiltration^27^. Then, the R packages ‘GSVA’ and ‘GSEABase’ were used to perform immune function analysis on the high- and low-risk groups^31^.

### Gene mutation, tumour mutation burden (TMB), microsatellite instability (MSI) status and immunotherapy analysis

We further downloaded the CRC simple nucleotide variation data from the TCGA GDC database (https://portal.gdc.cancer.gov/) and then used the R package ‘maftools’ to analyse the gene mutations in the high- and low-risk groups and draw a waterfall diagram^32^. Furthermore, we analysed the relationship between the two risk groups and MSI. Then, immunotherapy response data were downloaded from the TIDE website (http://tide.dfci.harvard.edu/). Using R packages ‘limma’, we analysed the relationship between risk score and immunotherapy response in CRC.

### Development and validation of a nomogram including DSP risk score and clinical characteristics

Univariate and multivariate Cox analyses were used for selected independent prognostic factors. Further, clinical characteristics and DSP risk scores were used to build a prediction nomogram using the R package ‘rms’ (available from: http://CRAN.R-project.org/package=rms). The ‘timeROC’ package was used to construct the ROC curve and calculate the AUC^33^. Calibration plots of the nomogram depicted the predictive value between the predicted 1-, 3-, and 5-year survival events and virtually observed outcomes^34^.

### Statistical analyses

All statistical analyses were performed using R version 4.2.2. Pearson correlation analysis was conducted to examine the correlation between two variables. The Wilcoxon test was used to analyse the difference of immune cell abundance among different groups. The Kaplan–Meier method (Log-rank test) was used for survival analysis. Univariate and multivariate Cox regression analyses were performed to analyse the prognostic factors of patients with CRC. Statistical significance was set at *P* < 0.05.

## Results

### DSP molecular subtypes in CRC

**Figure 1:**
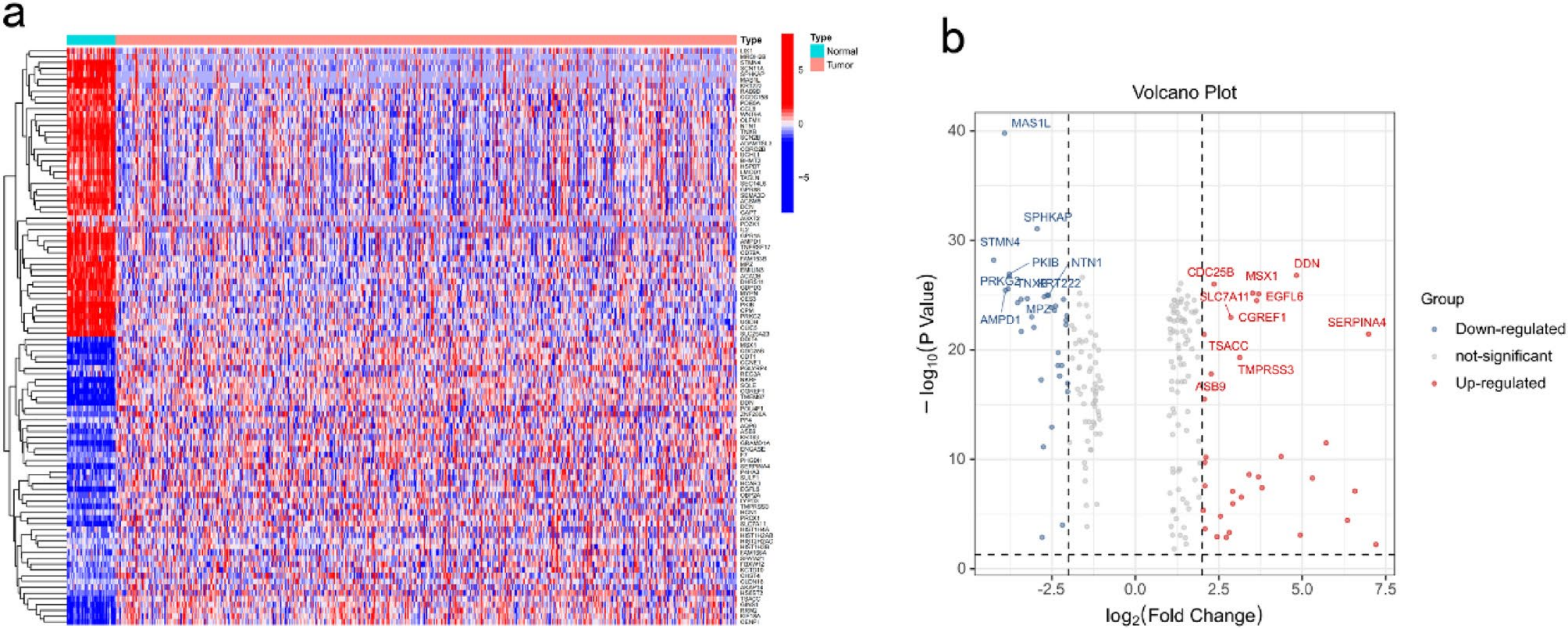
Differential expression of DSP-related genes in colorectal cancer and normal tissues. (**a**) Heat map and (**b**) volcano map showing the differential expression of DSP-related genes in colorectal cancer and normal tissues. DSP, disulfidptosis.

**Figure 2:**
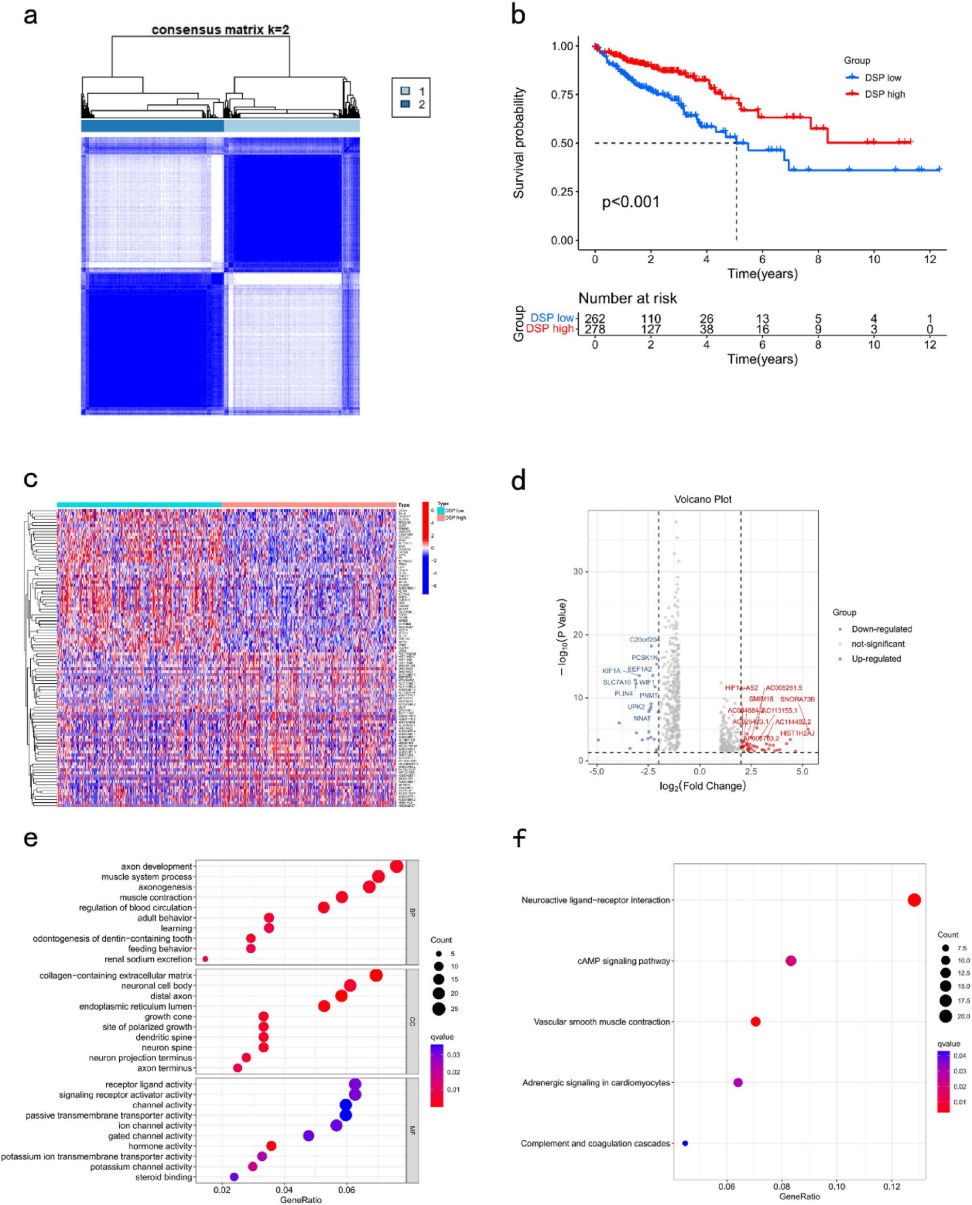
Identification and analysis of DSP-related gene subtypes in colorectal cancer. (**a**) Unsupervised consensus cluster analysis identified the best DSP-related gene subtypes in colorectal cancer as k = 2. (**b**) Kaplan–Meier analysis of survival prognosis of two DSP subtypes. (**c**) Heat map and (**d**) volcano map display of DEGs and the identification of two DSP subtypes. GO (**e**) and KEGG (**f**) analysis about DSP-related DEGs. DSP, disulfidptosis; DEGs, differential expression genes.

A total of 213 DSP-related genes were differentially expressed in cancer and normal tissues (Fig. 1a,b; Table S2). Unsupervised consistent clustering results showed that k = 2 was the best choice for dividing all patients into two subtypes (Fig. 2a, Fig. S2). The DSP high subgroup had a longer OS than those in the low-DSP subgroup (Fig. 2b).

### Characteristics of clinical DSP subtypes and the tumour immune microenvironment

**Figure 3:**
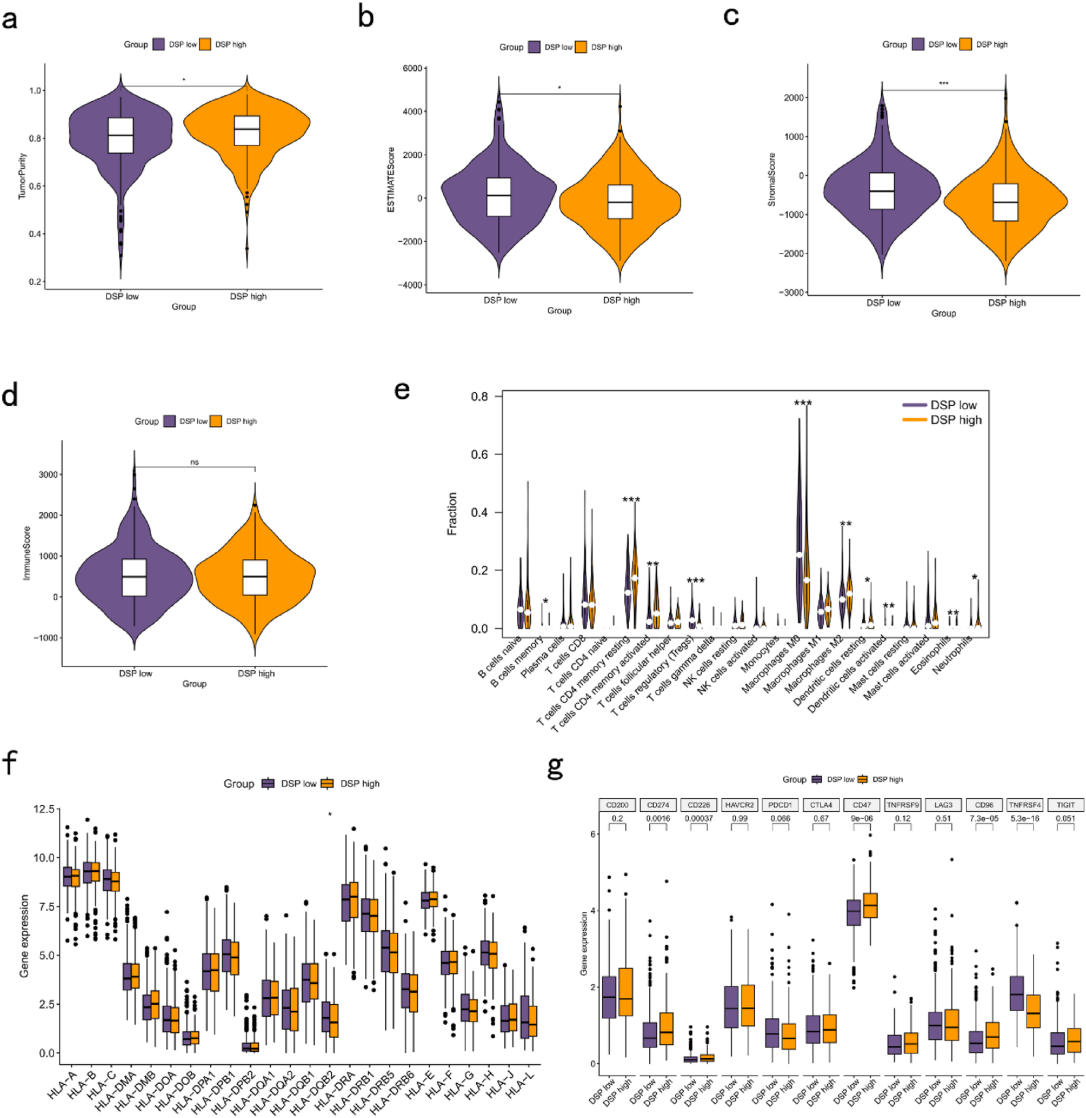
Correlation analysis between DSP subtypes and immune microenvironment. ESTIMATE analysis of DSP subtypes in (**a**) tumour purity, (**b**) estimate score, (**c**) stromal score, and (**d**) immune score. (**e**) CIBERSORT analysis of immune cell infiltration abundance in DSP subtypes. (**f**) Analysis of differential expression of HLA-related genes in DSP subtypes. (**g**) Expression of immune checkpoints in differential DSP subtypes. DSP, disulfidptosis. *P < 0.05, **P < 0.01, and ***P < 0.001.

DEGs were identified in the two DSP molecular subtypes (Fig. 2c,d). Functional enrichment analysis showed that DEGs in the DSP subtypes were enriched in neuroactive ligand-receptor interactions and axon development (Fig. 2e,f). ESTIMATE analysis results suggested that the stromal (*P* < 0.001) and estimate (*P* < 0.05) scores in the DSP-low group were markedly higher than those in the DSP-high group, whereas the tumour purity in the DSP-low group was lower than that in the DSP-high group (Fig. 3a–d). Additionally, CIBERSORT results showed that the numbers of CD4+ T cells, M2 macrophages, dendritic cells, and neutrophils were significantly higher in the DSP-high group than in the DSP-low group (Fig. 3e). The expression of HLA-DQB2 was higher in the low-DSP group than in the high-DSP group (Fig. 3f), whereas CD274 expression was higher in the high-DSP group (Fig. 3g).

### A novel DSP-related gene prognostic signature

**Figure 4:**
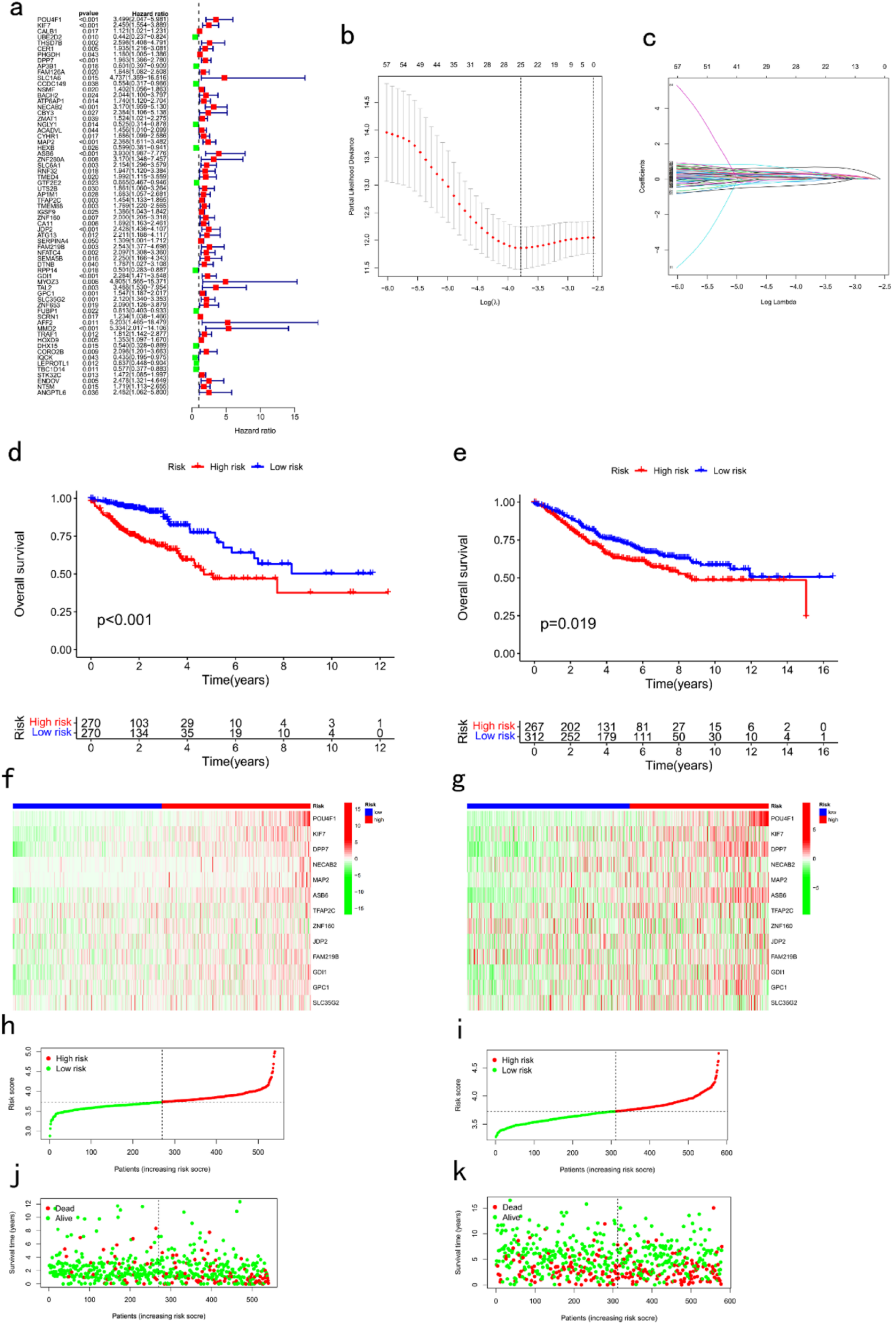
Construction and evaluation of a DSP prognostic signature for CRC. (**a**) Univariate Cox analysis about DSP-related genes differential expression in colorectal cancer and normal tissue. (**b, c**) LASSO regression analysis of DSP-related genes which are most significantly correlated with OS. Survival analysis of CRC patients in the (**d**) training and (**e**) testing datasets. Signature gene expression in the high- and low-risk groups in the (**f**) training and (**g**) testing datasets. Risk scores of patients in the high- and low-risk groups in the (**h**) training and (**i**) testing cohorts. Survival status of each patient in the (**j**) training and (**k**) testing datasets. DSP, disulfidptosis. LASSO, least absolute shrinkage and selection operator; OS, overall survival; CRC, colorectal cancer. *P < 0.05, **P < 0.01, and ***P < 0.001.

A total of 57 DSP prognostic genes were selected for subsequent analysis (*P* < 0.05; Fig. 4a). For prognostic signature construction, 13 genes were utilized (POU4F1, KIF7, DPP7, NECAB2, MAP2, ASB6, TFAP2C, ZNF160, JDP2, FAM219B, GDI1, GPC1, and SLC35G2; Fig. 4b,c). Risk score = (0.512 × expression of POU4F1) + (0.176 × expression of KIF7) + (0.142 × expression of DPP7) + (0.038 × expression of NECAB2) + (0.171 × expression of MAP2) + (0.129 × expression of ASB6) + (0.009 × expression of TFAP2C) + (0.012 × expression of ZNF160) + (0.039 × expression of JDP2) + (0.003 × expression of FAM219B) + (0.048 × expression of GDI1) + (0.002 × expression of GPC1) + (0.010 × expression of SLC35G2). The OS of the patients in the training and testing datasets with high-risk scores was significantly worse than that of the patients with low-risk scores (*P* < 0.05; Fig. 4d,e). The heat map shows the expression of the signature genes in the high- and low-risk groups in the training and testing datasets (Fig. 4f,g). In addition, we ranked the patient risk scores and analyzed their distribution in the training and testing sets (Fig. 4h–k).

### DSP risk score and clinicopathological characteristics

**Figure 5:**
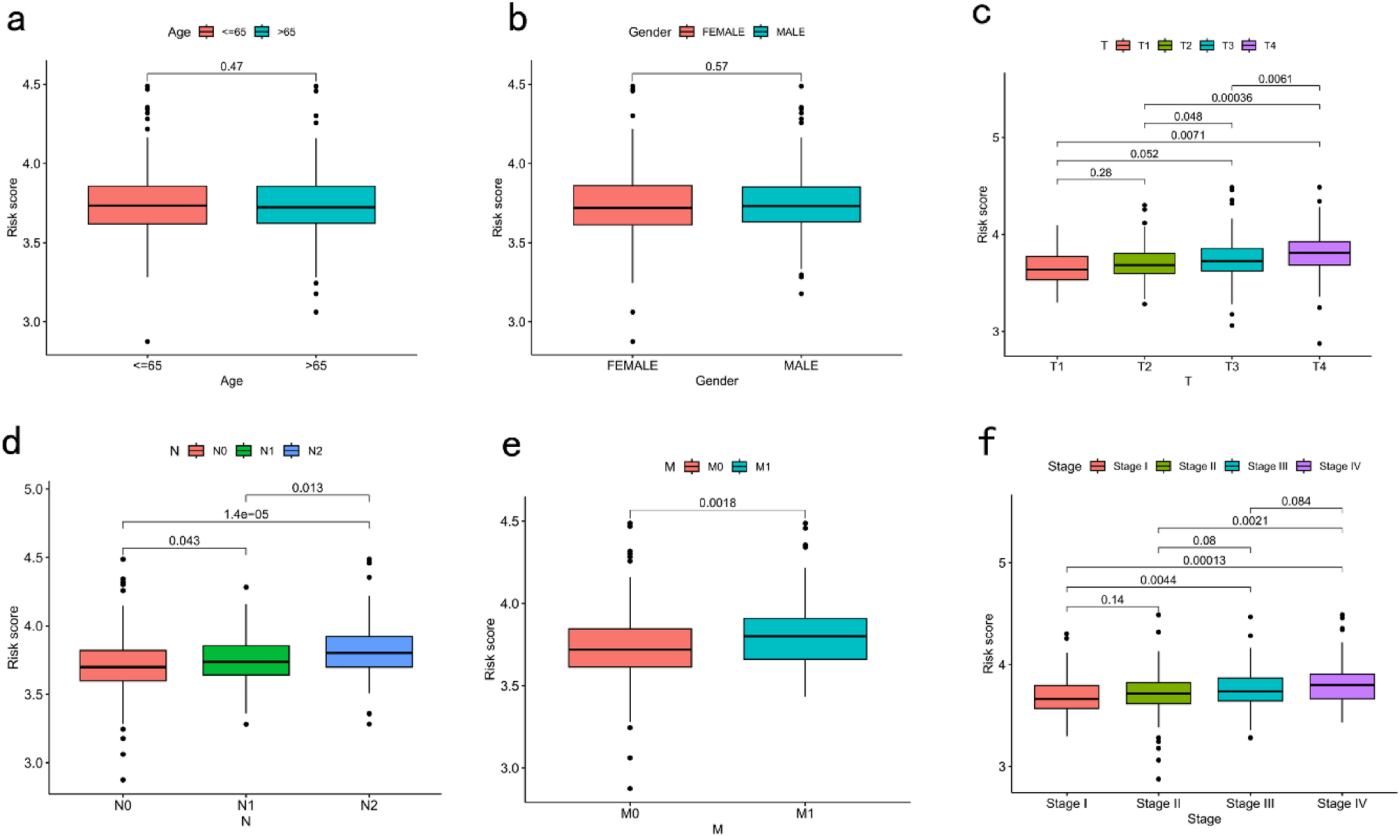
Relationship between risk score and clinicopathological characteristics in CRC. Correlations of risk scores and (**a**) age, (**b**) gender, (**c**) T stage, (**d**) N stage, (**e**) M stage, and (**f**) TNM stage. CRC, colorectal cancer.

High risk scores were more likely to be associated with higher T-, N-, and M-staging as well as total staging, regardless of age or sex (Fig. 5a–f).

### Immune microenvironment in the DSP prognostic signature

**Figure 6:**
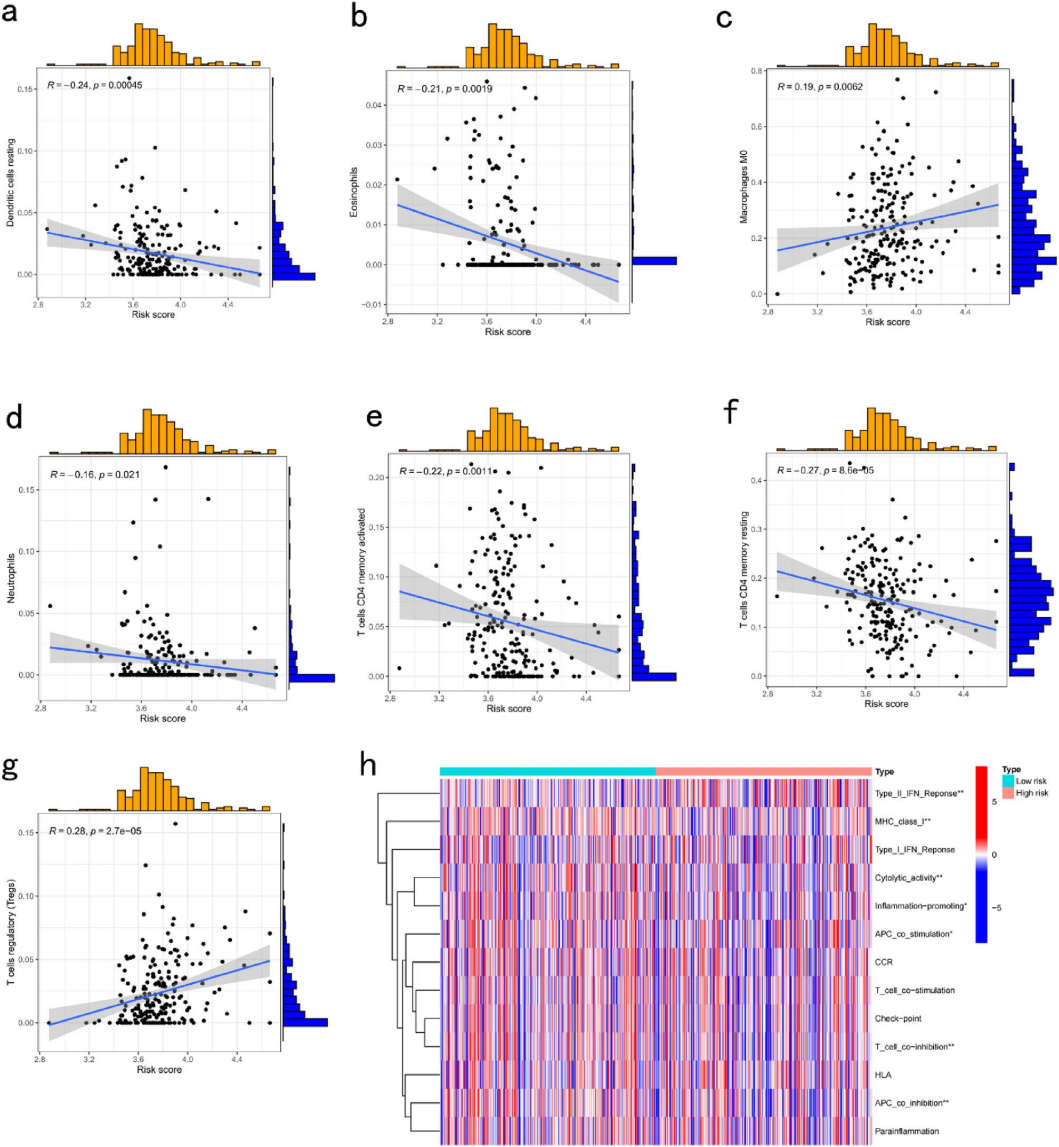
Relationship between risk score and immune microenvironment in CRC. Analysis of risk score and (**a**) dendritic cell resting, (**b**) eosinophils, (**c**) macrophage M0, (**d**) neutrophils, (**e**) T cells CD4+ memory activated, (**f**) T cells CD4+ memory resting, and (**g**) T cells regulatory (Tregs). (h) Analysis of differences in immune function between the high- and low-risk groups. CRC, colorectal cancer. *P < 0.05, **P < 0.01, and ***P < 0.001.

Immune cell infiltration analysis revealed that risk scores negatively correlated with dendritic cell, eosinophil, and CD4+ T cell numbers and significantly positively correlated with Treg cell and M0 macrophage numbers (Fig. 6a–g). Immune function analysis showed that cytolytic activity, inflammation promotion, and APC co-stimulation were significantly enriched in the low-risk group (Fig. 6h).

### Prognostic signature guided the clinical treatment decisions of patients with CRC

**Figure 7:**
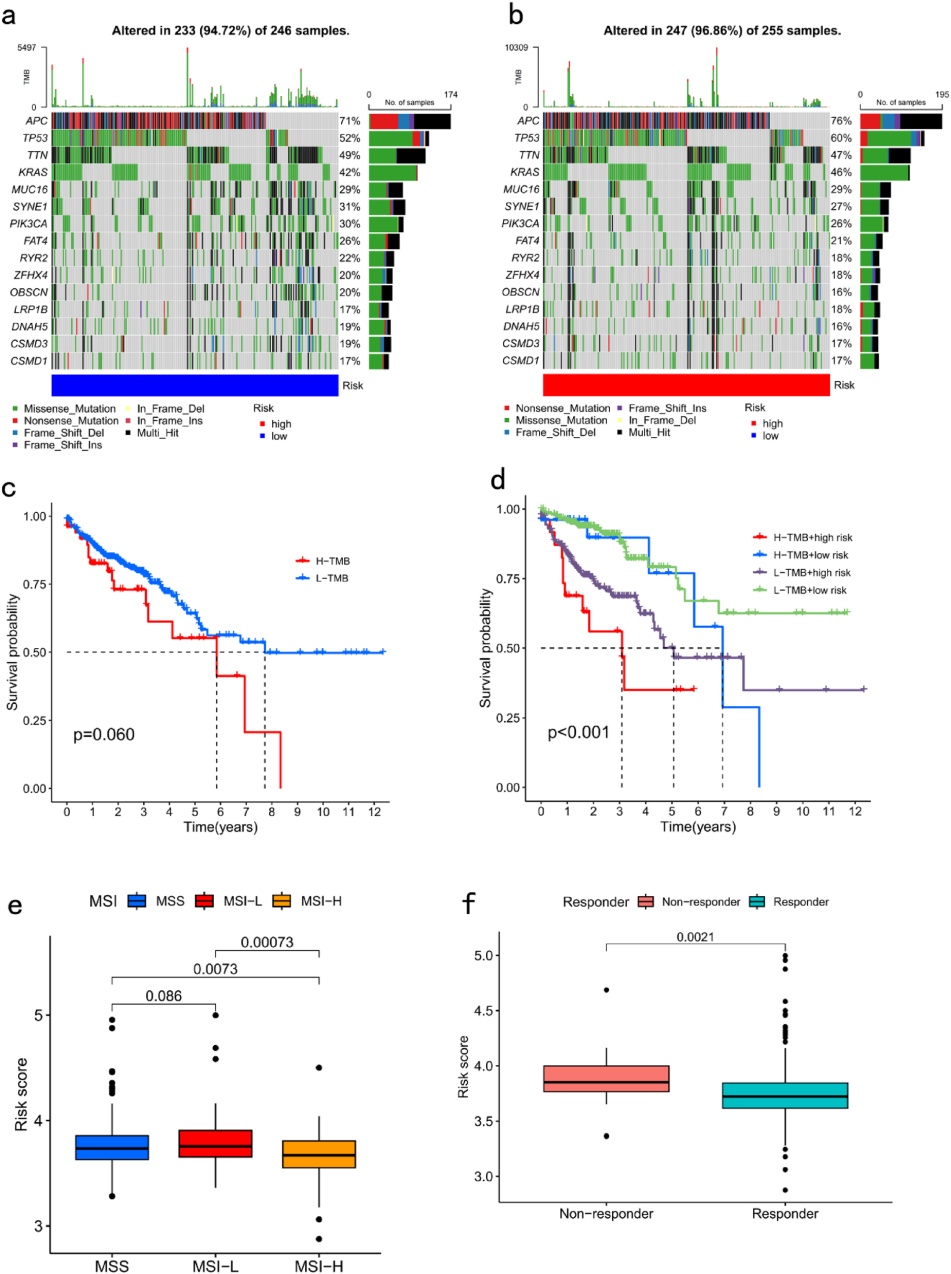
Gene mutation frequency, tumour mutation burden (TMB), MSI status, and immune escape analysis in the risk-prognostic signature. Analysis of gene mutation frequencies in the (**a**) low- and (**b**) high-risk groups. (**c**) Survival analysis of TMB. (**d**) Survival analysis of TMB combined with risk score. (**e**) Relationship between MSI status and risk score. (**f**) Analysis of differences in risk scores for different immunotherapy responses.

Gene mutation analysis showed that the frequency of gene mutations was higher in the high-risk group than in the low-risk group (Fig. 7a–b). The TMB survival analysis revealed poorer survival among the patients in the H-TMB group than those in the L-TMB group, although the p value was not statistically significant (Fig. 7c). The survival of the patients in the H-TMB group was significantly worse than that of the patients in the L-TMB group (Fig. 7d). The risk score was significantly lower in the MSI-H group than in the MSS and MSI-L groups (Fig. 7e). TIDE (http://tide.dfci.harvard.edu/) represents tumor immune dysfunction and rejection. In our TIDE-based study, the patients with high-risk scores were more prone to being non-responsive to immunotherapy than those with low-risk scores (*P* = 0.0021; Fig. 7f).

### Development of a nomogram to predict survival

**Figure 8:**
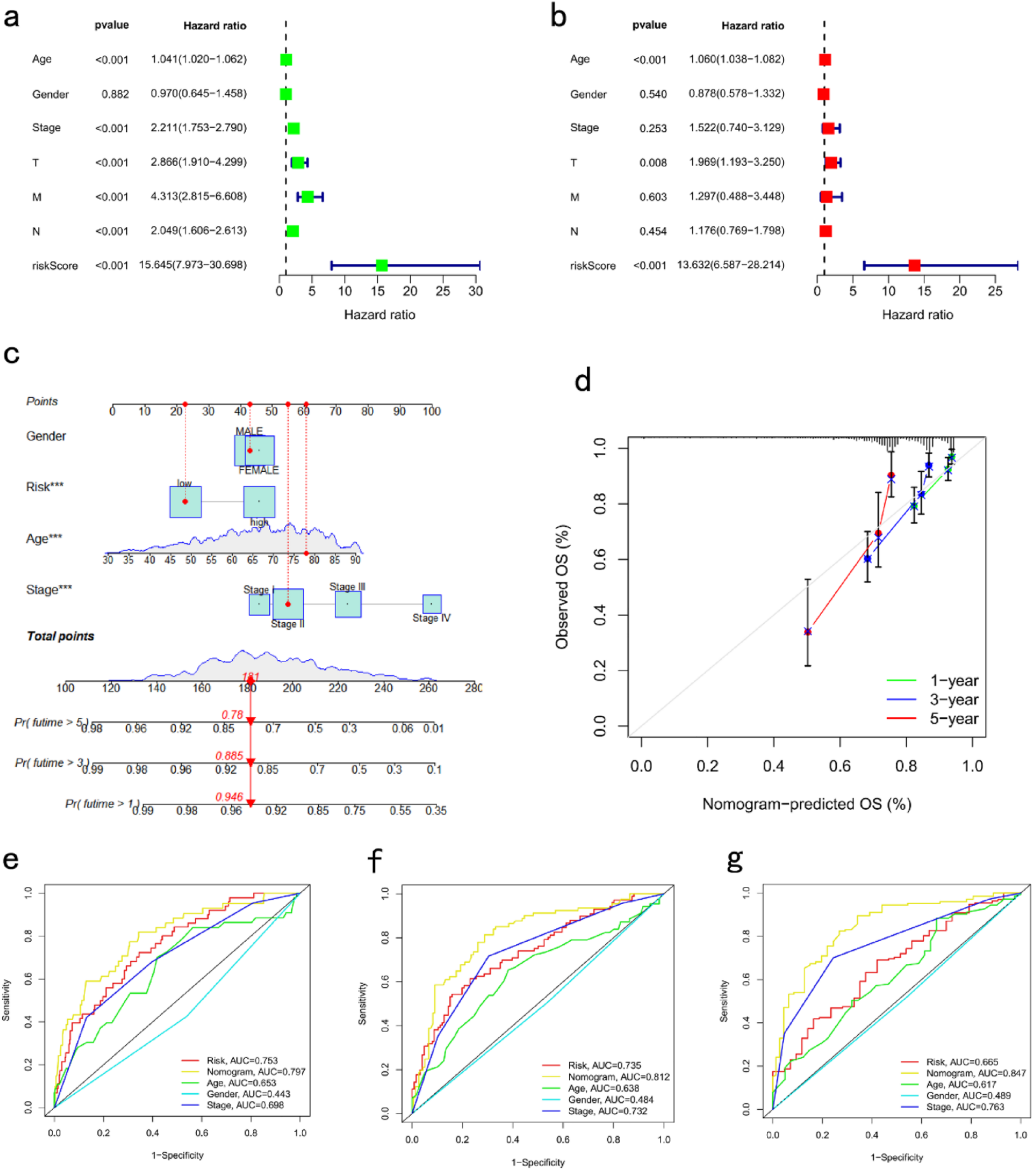
Establishment of a nomogram signature based on clinicopathological characteristics and risk scores. Forest plots based on (**a**) univariate and (**b**) multivariate Cox regression analyses for overall survival (OS). (**c**) Nomogram established based on risk score and clinicopathological features. (**d**) Calibration curves of the nomogram for predicting the 1-, 3-, and 5-year OS rates. 1-year (**e**), 3-year (**f**), and 5-year (**g**) ROC analysis for nomogram, risk score, and clinicopathological features. *P < 0.05, **P < 0.01, and ***P < 0.001. OS, overall survival; ROC, Receiver operating characteristic curve.

We analyzed the association between the risk scores and clinicopathological characteristics and found that the risk score was an independent predictor of OS (Fig. 8a,b; *P* < 0.001). A novel nomogram containing the risk scores and clinicopathological characteristics was successfully constructed (Fig. 8c). The calibration curves of the nomogram for predicting the 1-, 3-, and 5-year OS rates suggested that the performance of the proposed nomogram was similar to that of the current ideal model (Fig. 8d). In predicting the survival prognosis of patients, the nomogram 1-, 3-, and 5-year ROC AUC (0.797, 0.812, and 0.847, respectively) were better than the DSP-related risk score and stage distribution (Fig. 8e–g). The predicted clinicopathological characteristics were consistent with previous literature analyzed by TCGA COAD/READ cohort^35^.

## Discussion

This study comprehensively analysed DSP-related genes, identified two different DSP subtypes in colorectal cancer, and analysed the differences between the two DSP subtypes in terms of function, immune microenvironment, and survival prognosis. A DSP-related prognostic signature in the colorectal cancer TCGA cohort was constructed and validated using an external cohort. A nomogram, including clinical characteristics and risk prognostic model scores, was constructed and shown to perform well. Finally, we determined that the DSP prognostic model risk score was correlated with immunotherapy response and tumour microenvironment.

DSP involves multiple mechanisms. Accumulating evidence indicates that DSP plays an important role in biological processes and has been associated with the development and metastasis of malignant tumours for decades. We created a signature of 13 DSP-associated genes (POU4F1, KIF7, DPP7, NECAB2, MAP2, ASB6, TFAP2C, ZNF160, JDP2, FAM219B, GDI1, GPC1, and SLC35G2) and found that it can predict OS in patients with CRC. POU4F1 is a stem cell-associated transcription factor, whose overexpression contributes to the acquired resistance of melanoma cells to vemurafenib^36^. It is a potential therapeutic target in combination with BRAFi therapy for melanoma. KIF7 is a kinesin-4 family member that plays a critical role in Hedgehog (Hh) signalling during embryonic development. Further, Yao et al.^37^ found that low KIF7 expression indicates poor prognosis in epithelial ovarian cancer. Li et al.^38^ have reported that KIF7 interacts with Sufu to inhibit basal cell carcinoma in vivo. DPP7 is a member of the dipeptidyl peptidase family of proteins, which is highly expressed in breast cancer and is associated with a better prognosis^39^. N-terminal EF-hand calcium-binding protein 2 (NECAB2) is mainly involved in the regulation of calcium homeostasis in neurons, and only a few studies focused on NECAB2 expression in tumours. Lee et al.^40^ found that NECAB2 is highly expressed in HCC tissues and could be used as a prognostic factor. MAP2, which belongs to the family of microtubule-associated proteins, is expressed at low levels in NSCLC and is associated with better survival^41^. Huang et al.^42^ found that ASB6 attenuates ER stress to increase the stem cell characteristics of oral squamous cell carcinoma cells and enhance their metastatic ability. The transcription factor TFAP2C is involved in tumour development and chemotherapy sensitivity^43^ and has potential as a biomarker of treatment resistance in colorectal cancer^44^. The transcriptional modulator Jun dimerization protein 2 (JDP2) is closely related to tumour differentiation and apoptosis and participates in the regulation of CD8+ T cell immune function^45^. GDI1, a subtype of the GDP dissociation inhibitor, regulates the GDP/GTP exchange reaction of the Rab family. High GDI1 expression was significantly associated with poor prognosis of patients with CRC. Thus, GDI1 can be used as a prognostic biomarker for CRC^46^. Glypicans (GPCs) are a family of heparan sulphate proteoglycans (HSPGS). GPC1 promotes the proliferation and migration of colorectal cancer cells^47^. In our study, all genes in the risk-prognostic model were risk factors for the prognosis of patients with CRC.

The tumor microenvironment can mediate immune escape to promote tumour occurrence and development. In the present study, two distinct subtypes of DSP in CRC were identified. The expression level of CD274 was significantly higher in the DSP-high group than in the DSP-low group. Many tumour cells (including melanoma and non-small cell lung cancer) can overexpress PD-L1^48^, and after binding to PD-1 on the surface of T cells, it inhibits the proliferation and differentiation of T cells and reduces the secretion of cytokines, resulting in the loss of the tumour-killing function of T cells. Our study showed that the OS of the DSP-high group was significantly lower than that of the DSP-low group, which may be due to tumour immune escape. We constructed a prognostic risk model based on DSP-related gene expression. Differences in immune cell infiltration between the high- and low-risk groups were further analysed. Dendritic cell, eosinophil, and CD4+ T cell numbers were negatively correlated with the risk score, while Treg cells were positively correlated with the risk score. Previous studies have shown that dendritic cells^49^, eosinophils^50^, and CD4+ T cells^51^ are mainly involved in antitumour immune responses and mediate tumour-killing effects, while Treg cells mainly lead to immune suppression. Our results are consistent with those of previous studies. In addition, the high-risk DSP group had a higher number of non-responder patients than those in the low-risk group, indicating that CRC in patients in the high-risk DSP group was more likely to be associated with immune system escape.

Although our prognostic signature showed good performance in the training and validation cohorts, this study still has some limitations. Firstly, the study was retrospective, which inevitably resulted in a certain degree of bias. Secondly, the biological functions of the DSP genes FAM219B and SLC35G2 in our prognostic signature have not been studied in CRC cells, which requires further experimental studies. Therefore, more high-quality, multicenter, randomized controlled trials with large sample sizes and sufficient follow-up are required for further verification.

## Conclusion

In this study, we found that DSP-related genes are closely related to the occurrence and development of CRC and participate in regulating the immune microenvironment, mediating the immune response to CRC. We constructed and validated a DSP-related gene prognostic signature which can effectively predict the survival prognosis of CRC patients. Our study preliminarily explored the relationship between DSP and CRC and laid the foundation for further research.

### Supplementary Information

Supplementary Information.

## Data Availability

The datasets analysed in the current study are available from The Cancer Genome Atlas (TCGA) repository (https://portal.gdc.cancer.gov/), cohort (TCGA-READ and TCGA-COAD), and the GEO database GSE39582 dataset.
