# Supplementary material for: Disulfidptosis-related prognostic signature correlates with immunotherapy response in colorectal cancer
Source: Sci Rep. 2024 Jan 2;14:81. doi: 10.1038/s41598-023-49954-w (PMC10762008; doi:10.1038/s41598-023-49954-w)
Supplement: Supplementary file 1 — Supplementary Information. [file 41598_2023_49954_MOESM1_ESM.docx]

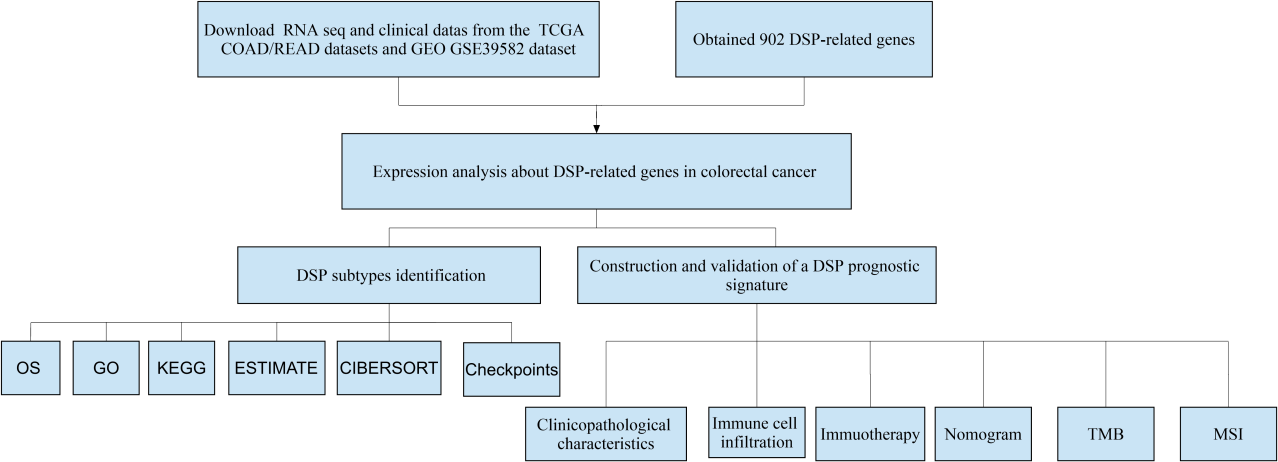


Figure S1. Flow chart of this research.


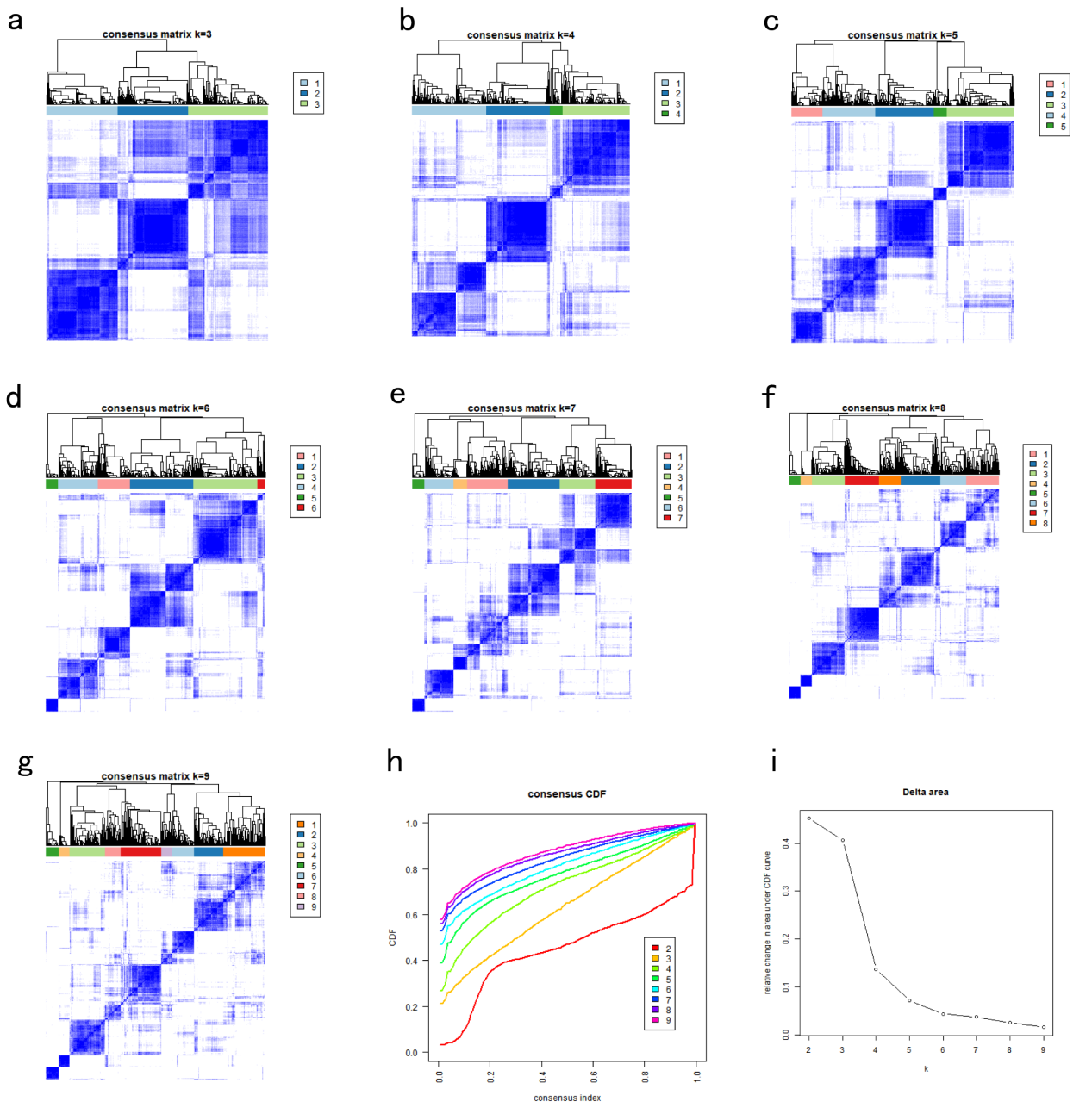


**Figure S2.** Identification of DSP subtypes in colorectal cancer. Consensus matrix heatmap defining (a) 3 clusters (k=3), (b) 4 clusters (k=4), (c)5 clusters (k=5), (d) 6 clusters (k=6), (e) 7 clusters (k=7), (f) 8 clusters (k=8), (g) 9 clusters (k=9). (h) Consensus clustering cumulative distribution function (CDF) for k = 2 to 9. (i) Relative changes in the areas under the CDF curve for k = 2 to 9. DSP, disulfidptosis.

**Table S1.** **Clinicopathological characteristics of patients with colorectal cancer in TCGA and GEO databases.**

|  | TCGA READ/COAD cohort | GEO GSE39582 cohort |
| --- | --- | --- |
| Patients(n) | 540 | 585 |
| Age(years)(n) |  |  |
| ≤65 | 235 | 228 |
| >65 | 305 | 356 |
| unknown | 0 | 1 |
| Gender(n) |  |  |
| Male | 287 | 322 |
| Female | 253 | 263 |
| T Stage(n) |  |  |
| Tis  T1 | 1  15 | 4  12 |
| T2 | 93 | 49 |
| T3 | 368 | 379 |
| T4 | 63 | 119 |
| unknown | 0 | 22 |
| N Stage(n) |  |  |
| N0 | 317 | 314 |
| N1 | 129 | 137 |
| N2 | 93 | 100 |
| N3  N+  unknown | 0  0  1 | 6  6  22 |
| M Stage |  |  |
| M0 | 401 | 499 |
| M1 | 76 | 61 |
| unknown | 63 | 25 |
| TNM Stage(n) |  |  |
| 0  Ⅰ | 0  93 | 4  38 |
| Ⅱ | 207 | 271 |
| Ⅲ | 148 | 210 |
| Ⅳ | 77 | 60 |
| unknown | 15 | 2 |
| Survival status(n) |  |  |
| Alive | 431 | 385 |
| Death | 109 | 194 |
| unknown | 0 | 6 |

**Table S2. Differential expression of disulfidptosis-related genes in colorectal cancer tissues and normal tissues.**

| gene | NormalMean | CancerMean | logFC | pValue | fdr |
| --- | --- | --- | --- | --- | --- |
| SLC7A11 | 0.41766816 | 3.041000881 | 2.864117161 | 1.09E-23 | 1.56E-22 |
| SLC3A2 | 17.01797645 | 40.77707817 | 1.260698903 | 4.12E-24 | 6.52E-23 |
| SKA1 | 1.54856517 | 4.05346987 | 1.38822532 | 8.59E-18 | 4.20E-17 |
| CPM | 11.74929034 | 2.246082017 | -2.387091106 | 9.67E-25 | 1.90E-23 |
| POU4F1 | 0.016356117 | 0.123260547 | 2.913808863 | 8.55E-08 | 1.64E-07 |
| LIX1 | 0.124491658 | 0.031838273 | -1.967215089 | 2.82E-12 | 7.60E-12 |
| SERPINE2 | 3.760793016 | 10.92303586 | 1.538265072 | 2.31E-13 | 7.04E-13 |
| KRT83 | 0.010188679 | 0.210783099 | 4.37072024 | 5.55E-11 | 1.36E-10 |
| EVI2B | 10.00965589 | 4.155634436 | -1.268251724 | 4.19E-17 | 1.83E-16 |
| FMO3 | 0.163693525 | 0.357482122 | 1.126873836 | 5.45E-06 | 8.95E-06 |
| NUTF2 | 16.01611636 | 35.48784622 | 1.147800658 | 4.12E-24 | 6.52E-23 |
| TMEM97 | 8.005333614 | 27.16146974 | 1.762528116 | 2.86E-25 | 8.01E-24 |
| FAM186A | 0.028586527 | 0.062025729 | 1.117531428 | 0.000822832 | 0.001138824 |
| AGXT2 | 0.069466391 | 0.010042348 | -2.790218563 | 0.001335946 | 0.001787809 |
| PDE5A | 7.559056545 | 2.367529524 | -1.674823765 | 1.32E-21 | 1.15E-20 |
| UGDH | 58.26142182 | 18.18517086 | -1.679778429 | 7.21E-25 | 1.59E-23 |
| HIST1H3B | 0.136008442 | 11.11036315 | 6.352065966 | 3.85E-05 | 5.87E-05 |
| SCN2B | 0.670266984 | 0.058212699 | -3.525330046 | 5.16E-25 | 1.21E-23 |
| GAPT | 0.844438625 | 0.269072581 | -1.649997185 | 1.04E-16 | 4.34E-16 |
| MROH2B | 0.029083449 | 0.009466434 | -1.619305354 | 1.52E-20 | 1.13E-19 |
| SLCO1C1 | 0.115728009 | 0.0527184 | -1.13435958 | 2.78E-17 | 1.26E-16 |
| SNX10 | 2.004168345 | 4.110482521 | 1.036304062 | 1.24E-07 | 2.35E-07 |
| ACACB | 4.43874175 | 1.400741702 | -1.663959828 | 6.61E-26 | 3.44E-24 |
| BHMT2 | 1.063878455 | 0.261491856 | -2.024495414 | 1.14E-17 | 5.36E-17 |
| SLC25A23 | 44.52997932 | 14.84375024 | -1.58492131 | 2.30E-27 | 2.40E-25 |
| RUVBL2 | 19.46699136 | 42.76462826 | 1.135388067 | 1.89E-22 | 1.97E-21 |
| SCN11A | 0.278879603 | 0.029633658 | -3.234333793 | 2.12E-25 | 7.36E-24 |
| OLFM1 | 2.469138255 | 0.811438568 | -1.605453839 | 1.92E-21 | 1.63E-20 |
| FAM163B | 1.230836751 | 0.174556845 | -2.817870591 | 5.35E-18 | 2.73E-17 |
| CXCL13 | 13.51926468 | 4.7742798 | -1.50166166 | 9.28E-09 | 1.94E-08 |
| POLR1D | 10.94857791 | 26.24697441 | 1.261407634 | 1.91E-23 | 2.49E-22 |
| CCT6A | 32.62959614 | 80.13406327 | 1.296234501 | 3.32E-26 | 2.20E-24 |
| WFDC1 | 2.949960455 | 1.176996012 | -1.325586183 | 1.37E-11 | 3.47E-11 |
| APBA1 | 1.263073702 | 0.548527555 | -1.203302823 | 1.23E-17 | 5.75E-17 |
| PHGDH | 3.553831818 | 12.25415885 | 1.785824144 | 7.25E-09 | 1.52E-08 |
| DPP7 | 18.63163932 | 39.82366373 | 1.095871337 | 7.65E-18 | 3.81E-17 |
| DDIT4 | 15.77751925 | 47.26000725 | 1.582749467 | 2.58E-18 | 1.35E-17 |
| KCTD19 | 0.009815151 | 0.041630475 | 2.084557731 | 2.61E-08 | 5.27E-08 |
| XPOT | 6.022578841 | 17.2390077 | 1.517223453 | 1.66E-24 | 3.10E-23 |
| LIPH | 32.68244405 | 14.9977844 | -1.123766485 | 2.35E-18 | 1.26E-17 |
| C16orf96 | 0.031387328 | 0.014674305 | -1.096890036 | 9.03E-15 | 3.15E-14 |
| SEMA3D | 0.667909577 | 0.192511425 | -1.794708736 | 2.59E-21 | 2.17E-20 |
| SIAE | 30.6437245 | 11.50315282 | -1.413562322 | 9.70E-24 | 1.47E-22 |
| CENPH | 2.729195425 | 7.687941904 | 1.494121731 | 2.96E-23 | 3.47E-22 |
| IL1RAP | 0.406746086 | 0.90800805 | 1.158576623 | 5.92E-15 | 2.12E-14 |
| GDPD3 | 21.79447041 | 5.262482514 | -2.050146708 | 7.67E-24 | 1.19E-22 |
| FBXW12 | 0.013064946 | 0.04912044 | 1.910622325 | 2.88E-07 | 5.30E-07 |
| PKIB | 38.76754925 | 2.83090172 | -3.775515967 | 1.20E-27 | 2.19E-25 |
| KLHL10 | 0.049924941 | 0.0224693 | -1.151805516 | 1.29E-06 | 2.24E-06 |
| ENGASE | 5.283021727 | 15.66102785 | 1.567743653 | 4.32E-15 | 1.57E-14 |
| PFDN2 | 35.10073455 | 78.43859688 | 1.160062507 | 2.41E-23 | 2.98E-22 |
| HIST1H3E | 4.07657268 | 1.961955385 | -1.055064502 | 4.63E-13 | 1.36E-12 |
| SLC52A2 | 21.21608541 | 51.88854138 | 1.290257493 | 7.73E-21 | 6.05E-20 |
| SLC39A5 | 66.05397382 | 30.32810754 | -1.12298988 | 3.91E-15 | 1.43E-14 |
| PRMT3 | 2.010137375 | 5.286000049 | 1.394882338 | 2.50E-23 | 3.03E-22 |
| NSMF | 9.78859525 | 23.54081847 | 1.265990741 | 8.52E-18 | 4.19E-17 |
| BACH2 | 0.410395361 | 0.181191287 | -1.179500844 | 4.60E-13 | 1.36E-12 |
| NECAB2 | 0.156747765 | 0.063365447 | -1.306676615 | 1.32E-13 | 4.08E-13 |
| SLC8A3 | 0.080344298 | 0.035425864 | -1.181392579 | 4.69E-22 | 4.27E-21 |
| FANCG | 3.653662068 | 7.65020491 | 1.066155187 | 2.98E-22 | 2.85E-21 |
| CHRNA5 | 0.621040305 | 1.347142101 | 1.117143233 | 5.35E-13 | 1.55E-12 |
| LMOD1 | 66.63539848 | 6.238204456 | -3.417086036 | 2.10E-22 | 2.16E-21 |
| FOSB | 19.13790904 | 7.024381458 | -1.445990116 | 0.000133317 | 0.000195675 |
| DCAF13 | 3.01216925 | 8.219399759 | 1.448230206 | 1.78E-23 | 2.36E-22 |
| OBP2A | 0.014407545 | 0.130285645 | 3.176781699 | 2.90E-07 | 5.32E-07 |
| FAM124A | 0.866731827 | 0.308335286 | -1.491085679 | 6.10E-10 | 1.37E-09 |
| ST6GALNAC3 | 0.65219485 | 0.283675536 | -1.20106131 | 3.75E-16 | 1.52E-15 |
| HCN1 | 0.041254759 | 0.224633841 | 2.444942834 | 0.001185608 | 0.001598376 |
| NOP16 | 5.939760159 | 14.09574848 | 1.246783504 | 2.48E-22 | 2.44E-21 |
| ASB9 | 0.842313234 | 4.061301602 | 2.269513431 | 1.58E-18 | 8.67E-18 |
| LYPD3 | 0.488142441 | 2.063612161 | 2.079797758 | 1.90E-10 | 4.44E-10 |
| TTLL2 | 0.133394585 | 0.294864839 | 1.144353694 | 3.90E-06 | 6.49E-06 |
| HIST1H4A | 0.05109058 | 1.567881108 | 4.939615054 | 0.000829966 | 0.001146518 |
| MYPN | 0.273209547 | 0.054656774 | -2.321535672 | 2.66E-19 | 1.58E-18 |
| ANTXR2 | 14.19514723 | 7.068664486 | -1.005888241 | 3.08E-17 | 1.38E-16 |
| LRRC7 | 0.085151794 | 0.036250128 | -1.232050822 | 4.33E-21 | 3.46E-20 |
| URB2 | 1.851194548 | 4.822948893 | 1.381459002 | 2.38E-25 | 7.62E-24 |
| PRKG2 | 2.696175318 | 0.195956008 | -3.782312598 | 2.09E-27 | 2.40E-25 |
| PELI2 | 3.691508205 | 1.774352358 | -1.056917831 | 6.35E-18 | 3.21E-17 |
| C12orf66 | 0.946067909 | 1.937579901 | 1.034240155 | 1.13E-21 | 1.00E-20 |
| NXT1 | 10.02106802 | 28.66171922 | 1.516088874 | 5.74E-22 | 5.16E-21 |
| MAP2 | 0.398304173 | 0.176718133 | -1.172420512 | 7.15E-17 | 3.03E-16 |
| SLC46A3 | 17.80068427 | 7.81285512 | -1.188010934 | 1.48E-19 | 9.20E-19 |
| KIF18A | 0.795790434 | 2.369417586 | 1.574072019 | 1.33E-21 | 1.15E-20 |
| WNT9A | 1.735037986 | 0.594899246 | -1.544249994 | 8.91E-16 | 3.45E-15 |
| FOXP4 | 11.93791091 | 26.28816548 | 1.138863074 | 3.92E-19 | 2.26E-18 |
| MSX1 | 0.348982748 | 3.976028576 | 3.510100503 | 6.61E-26 | 3.44E-24 |
| UCHL1 | 8.042037977 | 2.177176018 | -1.885103099 | 5.89E-20 | 3.86E-19 |
| RRP1 | 4.821004955 | 12.06092757 | 1.322935047 | 1.03E-23 | 1.50E-22 |
| INHBC | 0.012775282 | 0.02855107 | 1.160189674 | 0.015313803 | 0.018580748 |
| RPL36A | 13.39888002 | 28.70655536 | 1.09926781 | 4.13E-17 | 1.81E-16 |
| IL2 | 0.074784503 | 0.022641817 | -1.723749608 | 3.85E-17 | 1.71E-16 |
| IFI44 | 3.214810584 | 6.768944579 | 1.074197168 | 0.000129541 | 0.000190903 |
| ZNF280A | 0.001926682 | 0.101270902 | 5.715957787 | 3.22E-12 | 8.62E-12 |
| SPATA21 | 0.003594467 | 0.023776582 | 2.725691454 | 0.001360421 | 0.001817223 |
| F7 | 0.050180571 | 0.696370343 | 3.79465396 | 4.10E-08 | 8.08E-08 |
| SLC6A1 | 0.087147773 | 0.217861048 | 1.321872581 | 7.55E-10 | 1.68E-09 |
| PROX1 | 1.198870316 | 5.023083252 | 2.066897578 | 3.17E-16 | 1.29E-15 |
| CDC25B | 8.395467614 | 43.03231485 | 2.357737861 | 9.93E-27 | 8.03E-25 |
| RNF32 | 0.441087361 | 1.205353426 | 1.450319898 | 2.11E-19 | 1.29E-18 |
| CES3 | 35.13707525 | 8.339417262 | -2.074975629 | 5.17E-23 | 5.97E-22 |
| PDZK1 | 2.204028989 | 0.485053513 | -2.183927374 | 0.000102389 | 0.000152432 |
| LAX1 | 1.50041633 | 0.642817519 | -1.222881717 | 6.46E-14 | 2.08E-13 |
| PF4 | 1.094479125 | 4.4708817 | 2.030314934 | 4.63E-06 | 7.68E-06 |
| FAM166A | 0.017561359 | 0.053148767 | 1.597631724 | 3.27E-06 | 5.47E-06 |
| CCDC158 | 0.152149724 | 0.042681202 | -1.833819007 | 1.31E-22 | 1.41E-21 |
| NOB1 | 17.1646688 | 41.75618405 | 1.282547855 | 4.34E-25 | 1.07E-23 |
| SERPINE3 | 0.030470377 | 0.087615789 | 1.523783514 | 0.005544954 | 0.007032624 |
| YARS | 7.888776841 | 16.27581151 | 1.044855946 | 1.22E-22 | 1.33E-21 |
| AMPD1 | 2.097067598 | 0.141496558 | -3.889534501 | 3.69E-26 | 2.24E-24 |
| CLDN18 | 0.049175147 | 7.260672134 | 7.206029924 | 0.005979824 | 0.007557833 |
| DHRS11 | 32.30332768 | 7.294327916 | -2.146835827 | 2.51E-25 | 7.62E-24 |
| HS6ST2 | 0.293037118 | 3.777923806 | 3.688438282 | 4.00E-09 | 8.52E-09 |
| HIST2H2AC | 1.056442409 | 7.426443091 | 2.813457272 | 0.000497376 | 0.000703086 |
| DDN | 0.036229901 | 1.027844615 | 4.826297521 | 1.59E-27 | 2.32E-25 |
| RPS21 | 324.5455841 | 901.6962442 | 1.474220386 | 7.95E-18 | 3.94E-17 |
| ITGB8 | 0.721288225 | 1.766863875 | 1.292543117 | 1.16E-09 | 2.56E-09 |
| TNXB | 7.066986193 | 0.504306535 | -3.808722265 | 2.72E-26 | 1.98E-24 |
| TWIST2 | 1.781407857 | 0.74604105 | -1.255690942 | 4.10E-14 | 1.33E-13 |
| TSACC | 0.136316803 | 0.567019785 | 2.056435671 | 3.86E-22 | 3.56E-21 |
| SH2D2A | 1.603703289 | 3.511802883 | 1.130804625 | 2.47E-15 | 9.21E-15 |
| RAB9B | 1.211325477 | 0.297816247 | -2.024092198 | 6.78E-17 | 2.89E-16 |
| ZNF697 | 0.77881203 | 2.21360325 | 1.507049594 | 2.91E-21 | 2.41E-20 |
| CENPW | 11.4461415 | 29.3864023 | 1.360287398 | 2.16E-19 | 1.31E-18 |
| TMPRSS3 | 0.341389235 | 2.980957157 | 3.126286168 | 5.04E-20 | 3.36E-19 |
| HCAR3 | 0.072619929 | 0.549627402 | 2.920016514 | 1.11E-06 | 1.94E-06 |
| PGLYRP4 | 0.01704554 | 0.099682086 | 2.547939979 | 1.59E-05 | 2.52E-05 |
| IGSF9 | 10.52902035 | 3.637690085 | -1.533276668 | 2.47E-17 | 1.13E-16 |
| DIXDC1 | 4.898595 | 1.739758518 | -1.493480948 | 3.65E-14 | 1.20E-13 |
| TNFRSF17 | 11.59668397 | 1.349804418 | -3.102890045 | 9.90E-24 | 1.47E-22 |
| MPZ | 3.766646086 | 0.566224475 | -2.733834468 | 1.38E-25 | 5.31E-24 |
| SQLE | 6.910906682 | 25.10517067 | 1.861037629 | 4.60E-20 | 3.10E-19 |
| AKAP14 | 0.020953562 | 0.089093198 | 2.088119791 | 0.000228925 | 0.000330015 |
| RRP12 | 2.748704614 | 8.063689681 | 1.552688242 | 5.78E-25 | 1.31E-23 |
| HSPB7 | 8.803723248 | 1.552226306 | -2.503774884 | 1.18E-13 | 3.71E-13 |
| NCAPH | 3.31196232 | 8.836185269 | 1.415737408 | 2.16E-22 | 2.18E-21 |
| WFDC3 | 0.371854011 | 1.084693589 | 1.544479319 | 1.03E-06 | 1.82E-06 |
| GINS1 | 2.440570859 | 9.039786991 | 1.889070139 | 2.66E-23 | 3.17E-22 |
| SERPINA4 | 0.005396349 | 0.684507437 | 6.986938842 | 3.57E-22 | 3.33E-21 |
| STMN4 | 0.406560344 | 0.021461661 | -4.243635878 | 6.30E-29 | 1.53E-26 |
| NUP43 | 5.384299318 | 11.15218177 | 1.050495463 | 1.64E-23 | 2.26E-22 |
| PLSCR4 | 10.24622116 | 3.709933958 | -1.465626527 | 8.05E-25 | 1.72E-23 |
| P4HA3 | 0.196825368 | 0.73684193 | 1.904438988 | 2.21E-12 | 6.07E-12 |
| CD79A | 30.69060093 | 6.182376686 | -2.311563431 | 1.84E-20 | 1.33E-19 |
| STMN3 | 6.079666159 | 17.19696786 | 1.500090203 | 0.001479886 | 0.001973182 |
| PNPLA1 | 0.135118172 | 0.342253421 | 1.340843245 | 0.000872759 | 0.001201075 |
| DTNB | 0.67781812 | 1.71043001 | 1.33538896 | 6.18E-20 | 4.02E-19 |
| EGFL6 | 0.123377461 | 1.599347385 | 3.69633256 | 7.82E-26 | 3.80E-24 |
| SPHKAP | 0.037421326 | 0.004876106 | -2.940059321 | 8.76E-32 | 3.19E-29 |
| SRRM5 | 0.126172783 | 0.307386854 | 1.284654727 | 7.15E-14 | 2.28E-13 |
| CGREF1 | 0.608744339 | 7.55274957 | 3.633093596 | 3.17E-25 | 8.24E-24 |
| EBI3 | 2.972502309 | 1.28507346 | -1.209827098 | 1.42E-15 | 5.45E-15 |
| DCN | 48.21155273 | 15.04724251 | -1.679879766 | 3.79E-19 | 2.21E-18 |
| SYT5 | 0.238498714 | 0.094942263 | -1.328859141 | 4.93E-19 | 2.80E-18 |
| TGIF1 | 5.508582068 | 12.23804776 | 1.151620519 | 2.30E-24 | 4.19E-23 |
| KRT222 | 0.121527112 | 0.019611165 | -2.631531052 | 9.78E-26 | 4.45E-24 |
| REG3A | 12.13762673 | 128.9405606 | 3.409147897 | 2.52E-09 | 5.43E-09 |
| NTN1 | 5.036671545 | 0.825892146 | -2.608445355 | 1.09E-25 | 4.65E-24 |
| CCL8 | 7.019200239 | 1.463103537 | -2.262274794 | 2.50E-18 | 1.32E-17 |
| GPR88 | 0.16390942 | 0.035543716 | -2.205232331 | 2.66E-19 | 1.58E-18 |
| POLR1B | 2.930834409 | 7.528689618 | 1.361087324 | 8.36E-27 | 7.61E-25 |
| ZEB1 | 5.943133682 | 2.064675534 | -1.525308754 | 2.04E-10 | 4.72E-10 |
| DNMT1 | 5.67779325 | 12.01652275 | 1.081617259 | 1.15E-19 | 7.34E-19 |
| ART1 | 0.048136899 | 0.017429305 | -1.465628157 | 1.67E-06 | 2.87E-06 |
| GRAMD1A | 4.945653045 | 15.95125423 | 1.689436928 | 3.50E-24 | 5.92E-23 |
| KLF1 | 0.037592023 | 0.100571799 | 1.419727353 | 4.27E-07 | 7.79E-07 |
| CHST4 | 0.035743296 | 1.415367403 | 5.307360123 | 5.35E-09 | 1.13E-08 |
| SULF1 | 6.084523068 | 23.41164984 | 1.944010518 | 2.13E-10 | 4.93E-10 |
| SEC14L6 | 0.158536513 | 0.049122531 | -1.690358346 | 4.68E-19 | 2.68E-18 |
| RAB37 | 2.410305157 | 0.928977487 | -1.37550027 | 9.26E-19 | 5.11E-18 |
| AK5 | 0.229498728 | 0.100499053 | -1.191304248 | 1.13E-16 | 4.66E-16 |
| ATAD3A | 6.727873955 | 16.45449545 | 1.290259207 | 3.69E-21 | 2.98E-20 |
| TAGLN | 387.4926584 | 57.35650645 | -2.756139919 | 7.31E-12 | 1.90E-11 |
| HIST1H2AB | 0.02741034 | 2.622385068 | 6.580015511 | 8.01E-08 | 1.55E-07 |
| AFF2 | 0.049488474 | 0.019680453 | -1.330329156 | 1.35E-11 | 3.44E-11 |
| HSP90AB1 | 234.4400932 | 551.3489408 | 1.233746353 | 9.24E-25 | 1.87E-23 |
| MMD2 | 0.021636589 | 0.008995569 | -1.266186697 | 8.00E-16 | 3.12E-15 |
| RETNLB | 32.19967695 | 15.40820007 | -1.063347873 | 2.25E-14 | 7.54E-14 |
| RFWD3 | 4.473814955 | 9.98967278 | 1.158931837 | 3.03E-25 | 8.16E-24 |
| SMYD2 | 4.752957023 | 9.52659316 | 1.003135022 | 1.39E-23 | 1.94E-22 |
| CORO2B | 1.315810039 | 0.312640992 | -2.07337237 | 2.02E-23 | 2.58E-22 |
| TNFSF10 | 50.53551955 | 19.69741333 | -1.359291574 | 3.66E-20 | 2.51E-19 |
| CDT1 | 5.517757905 | 17.88596881 | 1.696674201 | 8.41E-21 | 6.44E-20 |
| MAS1L | 0.227152551 | 0.015040926 | -3.916696178 | 1.62E-40 | 1.18E-37 |
| HM13 | 11.08117909 | 28.51270137 | 1.363493331 | 4.42E-25 | 1.07E-23 |
| CDRT1 | 0.017522955 | 0.047968289 | 1.452834859 | 0.000170797 | 0.000248184 |
| ACSM5 | 0.333606821 | 0.058282549 | -2.517012915 | 1.41E-24 | 2.71E-23 |
| CLIC5 | 23.03645152 | 6.169561675 | -1.900678605 | 6.01E-23 | 6.83E-22 |
| CENPI | 0.64749747 | 2.277626398 | 1.814584658 | 2.69E-24 | 4.66E-23 |
| TMEM41A | 2.321223795 | 5.223826986 | 1.170221492 | 2.81E-25 | 8.01E-24 |
| HS3ST3A1 | 0.202791397 | 0.474244223 | 1.225633751 | 2.78E-05 | 4.33E-05 |
| GPR15 | 8.955208666 | 0.832917982 | -3.426480704 | 2.49E-25 | 7.62E-24 |
| STK3 | 1.005402859 | 2.034789739 | 1.017106028 | 1.03E-14 | 3.57E-14 |
| PLEKHO1 | 10.19265261 | 3.784834603 | -1.429227397 | 2.27E-12 | 6.20E-12 |
| POLE2 | 1.321997477 | 3.168964836 | 1.261292227 | 7.26E-17 | 3.05E-16 |
| STK32C | 1.712287705 | 3.753618997 | 1.132357091 | 1.58E-09 | 3.43E-09 |
| ADAMTSL3 | 2.021296743 | 0.245644528 | -3.040637133 | 8.85E-23 | 9.76E-22 |
| LSM7 | 22.90009095 | 56.98510389 | 1.315231514 | 5.60E-20 | 3.71E-19 |
| AEN | 3.139919341 | 8.533080898 | 1.442339226 | 1.72E-23 | 2.32E-22 |
| SND1 | 27.94273409 | 59.69425452 | 1.095118891 | 8.99E-25 | 1.87E-23 |
| KCNMB2 | 0.129875298 | 0.049616378 | -1.388238743 | 3.69E-14 | 1.21E-13 |
| NKRF | 1.679185155 | 4.976078991 | 1.56724807 | 1.16E-25 | 4.69E-24 |
| IQGAP2 | 16.03885723 | 5.785397257 | -1.471083422 | 1.45E-21 | 1.24E-20 |
| CD200R1 | 0.692975927 | 0.302484507 | -1.195943986 | 4.88E-16 | 1.93E-15 |
| AQP6 | 0.026123035 | 0.112982186 | 2.112700901 | 6.25E-11 | 1.53E-10 |
| CCNE1 | 1.732892795 | 5.962431683 | 1.782718425 | 3.87E-24 | 6.40E-23 |
| CTLA4 | 0.469068349 | 1.150213338 | 1.29403141 | 5.80E-08 | 1.13E-07 |
| TCEA3 | 41.7099275 | 16.58944889 | -1.330124844 | 2.44E-22 | 2.43E-21 |
| EMILIN3 | 0.460971331 | 0.086325336 | -2.416821081 | 2.47E-24 | 4.39E-23 |
| FAM186B | 0.067795593 | 0.147247299 | 1.118977765 | 7.18E-10 | 1.60E-09 |
| LPCAT3 | 9.722134705 | 4.527437167 | -1.102578504 | 1.02E-20 | 7.73E-20 |
| RRM2 | 8.382063848 | 25.82220373 | 1.623234713 | 1.81E-22 | 1.91E-21 |
